# Bioinformatics Analysis of the *Panax ginseng* Cyclophilin Gene and Its Anti-*Phytophthora cactorum* Activity

**DOI:** 10.3390/plants13192731

**Published:** 2024-09-29

**Authors:** Yu Zhao, Jiahong Lu, Yuming Wang, Kaiwen Hao, Zhimei Liu, Ge Hui, Tianxia Sun

**Affiliations:** Jilin Ginseng Academy, Changchun University of Chinese Medicine, Changchun 130117, China; cnzhaoyu1972@126.com (Y.Z.); ljh1298832427@163.com (J.L.); 18940393301@163.com (Y.W.); 18835401877@163.com (K.H.); 15843048017@163.com (Z.L.)

**Keywords:** *Panax ginseng*, cyclophilin, *Phytophthora cactorum*, bioinformatics, novel pesticides

## Abstract

In this paper, *Panax ginseng* cyclophilin (PgCyP) was successfully obtained through a genetic engineering technique. A bioinformatics method was used to analyze the physicochemical properties and structure of *PgCyP*. The results showed that *PgCyP* belongs to the *cyclophilin* gene family. The protein encoded by the *PgCyP* gene contains the active site of PPIase (R62, F67, and H133) and a binding site for *cyclosporine* A (W128). The relative molecular weight of PgCyP is 187.11 bp; its theoretical isoelectric point is 7.67, and it encodes 174 amino acids. The promoter region of *PgCyP* mainly contains the low-temperature environmental stress response (LTR) element, abscisic acid-responsive cis-acting element (ABRE), and light-responsive cis-acting element (G-Box). *PgCyP* includes a total of nine phosphorylation sites, comprising four serine phosphorylation sites, three threonine phosphorylation sites, and two tyrosine phosphorylation sites. *PgCyP* was recombined and expressed in vitro, and its recombinant expression was investigated. Furthermore, it was found that the recombinant *PgCyP* protein could effectively inhibit the germination of *Phytophthora cactorum* spores and the normal growth of *Phytophthora cactorum* mycelia in vitro. Further experiments on the roots of susceptible *Arabidopsis thaliana* showed that the *PgCyP* protein could improve the resistance of arabidopsis to *Phytophthora cactorum*. The findings of this study provide a basis for the use of the *PgCyP* protein as a new type of green biopesticide.

## 1. Introduction

To survive in different environments, plants have evolved two innate immune systems to detect and cope with different biotic invasions [1]. These responses are initiated by pattern-recognition receptors (PRRs) located on the cell surface, leading to pattern-triggered immunity (PTI). Furthermore, intracellular nucleotide-binding domain leucine-rich repeat containing receptors (NLRs) activate effector-triggered immunity (ETI) [2]. PTI and ETI involve different steps in early signaling [3,4], yet they lead to many overlapping downstream outputs, such as calcium flux, reactive oxygen species (ROS) burst, transcriptional reprogramming, and phytohormone signaling [5,6]. NLRs can be divided into three types according to their different N-end domains: the coiled coil (CC) type, the Toll/INTERLEUKIN-1 receptor/resistance protein (TIR) type, and the RPW8 (CCR) type [7].

Antimicrobial peptides, one of the chemical defense factors, have been isolated and purified [8,9]. Antimicrobial peptides are small molecular peptides that resist the invasion of pathogens into cells and eliminate pathogens. The antimicrobial peptides recently isolated from plants mainly include ribosome-inactivating proteins, cyclophilin proteins (CyPs), protease inhibitors, defensins, and lipid transfer proteins, all of which exhibit bacteriostatic or bactericidal activity [10,11,12,13].

Cyclophilin can specifically bind to the immunosuppressant cyclosporine A and has peptidylproline cis–trans isomerase (PPIases) activity. There are two types of protein structures in the CyPs family: the first is the single domain (SD) or cyclophilin-like domain (CLD), which contains approximately 109 amino acid residues; the second is the multi-domain (MD), which contains the XXGKXLH sequence, one conserved glutamic residue, and two cysteine residues. The CyP gene is distributed in plant tissues and organs and plays an important role in the plant stress response and insect resistance response [14]. Cyclophilinases in potatoes are involved in many important defense reactions [15]. The CyP protein of the seeds of black-eyed peas can inhibit the growth of Botrytis cinerea [16]. The overexpression of an endogenous gene called *OsCYP*2 enhances the salt tolerance of rice seedlings [17]. The *CyP* gene of wheat is associated with the disease resistance of the tufted wheat 6VS/6AL translocation line [18]. The CyP protein of Chinese cabbage can inhibit the growth of pathogens [19]. The CyP gene regulates the activity of protein phosphatase 2A, thereby regulating many signaling pathways of the cell cycle, protein deficiency, reverse transcription, growth, and development [20].

*Panax ginseng* (*P. ginseng*) is prone to various root diseases caused by fungi and bacteria, with *Phytophthora cactorum* being one of the most destructive pathogens responsible for foliar blight and root rot [21]. The symptoms of foliar blight are dark green, water-soaked lesions on the leaves and root rot, leading to the wilting and reddening of the leaflets and brown spongy roots [22].

Currently, ginseng disease caused by *Phytophthora cactorum* (*P. cactorum*) is controlled by chemical pesticides. Unfortunately, the inappropriate use of chemical pesticides may induce pesticide resistance in *P. cactorum* [23,24], and pesticide residues aggravate pollution levels in the atmosphere, water, and food, thereby impacting biological diversity and human health [25]. Therefore, it is very urgent to develop a novel biological pesticide with low toxicity, high efficiency, and strong selectivity for the control of *P. cactorum*. There are few reports on the role of the CyP protein in inducing resistance in ginseng plants against *P. cactorum*. Furthermore, we successfully constructed the *P. ginseng* transcriptome database and analyzed the bioinformatics information of the CyP protein. Additionally, we examined whether the CyP protein is resistant to *P. cactorum*, and the results showed that the CyP protein has good resistance to *P. cactorum*. Our findings provide a theoretical and preliminary experimental basis for the development of a novel environmentally friendly biological pesticide with low toxicity and high efficiency.

## 2. Results

### 2.1. Cloning and Sequencing of the PgCyP Gene

**Figure 1:**
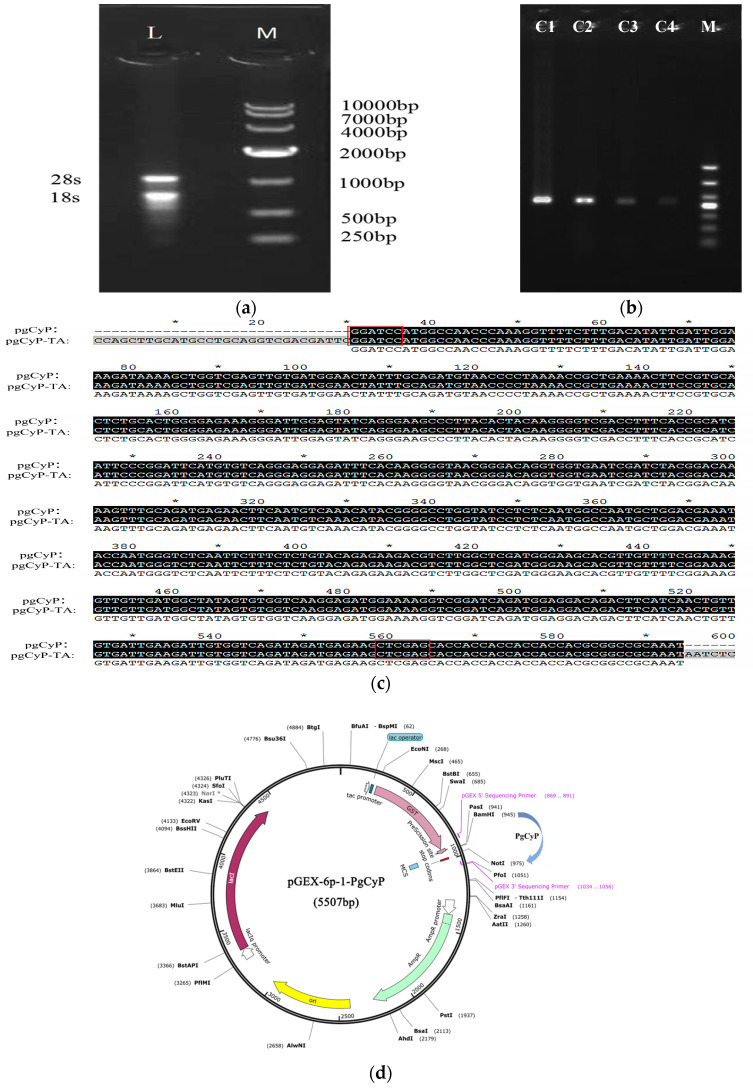
Cloning and sequence analysis of the *PgCyP* gene: (**a**) agarose gel electrophoresis of the total RNA extracted from ginseng; (**b**) PCR electrophoresis of the cloning vector; (**c**) results of sequence alignment analysis between the cloned plasmid and others(* Represents markers per 10 bases); and (**d**) plasmid construction diagram(* Blocked by Dcm methylation).

The *PgCyP* gene (ANA12002.1) (https://www.ncbi.nlm.nih.gov/protein/ANA12002.1?report=genbank&log$=protalign&blast_rank=1&RID=KXU8A9FP016) (accessed on 25 May 2024) was cloned by Zhang [26]. The molecular formula of the CYP protein in *P. ginseng* is C_825_H_1290_N_226_O_251_S_10_, and the CyP protein consists of a 525 bp coding sequence (CDS) that encodes 174 amino acids. As shown in Figure 1a,b, RNA was extracted and successfully transferred to competent cells. The gene was approximately 500 bp, similar to the *CyP* gene, and its sequence was found to match that of the *CyP* gene (Figure 1c); the plasmid mapping is shown in Figure 1d. The complete DNA sequence obtained was linked to the T vector, and then enzyme digestion was performed. The enzyme product was subsequently linked to the expression vector pGEX-6p-1, and the linked product was transferred into BL21 (DE3) for induced expression and purification; finally, the recombinant protein PgCyP was obtained.

### 2.2. Results of the Bioinformatics Analysis

#### 2.2.1. Analysis of Amino Acid Residues

**Figure 2:**
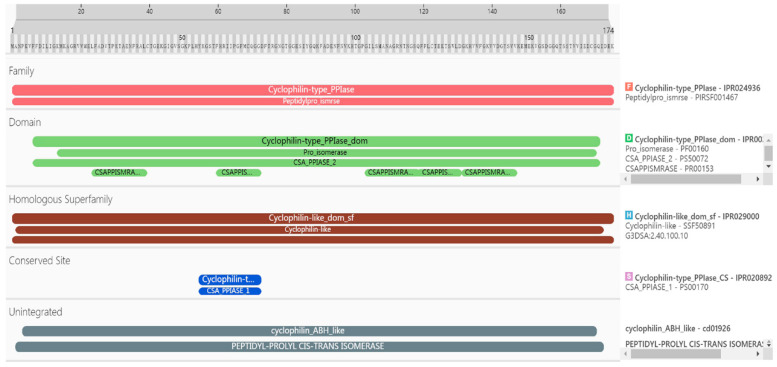
Full-length CDS and deduced amino acid sequence of the *PgCyP* gene in *P. ginseng*.

The details of the PgCyP protein domain analysis revealed that it belonged to the Cyclophilin-type_PPIase (IPR024936) family, the Peptidylpro_ismrse (PIRSF001467) family, the homologous superfamily, Cyclophilin-like_dom_sf (IPR029000), G3DSA (2.40.100.10), and (SSF50891). Furthermore, it included Cyclophilin-type_PPIase_domI (PR002130, 7aa-170aa) and Pro_isomerase (PF00160, 14aa-169aa); CSA_PPIASE_2 (PS50072, 7aa-170aa); and CSAPPISMRASE (PR00153, 24aa-39aa, 60aa-72aa, 103aa-118aa, 118aa-130aa, 131aa-146aa) structures. The conserved sites were Cyclophilin-type_PPIase_CS (IPR020892, 55aa-72aa) and CSA_PPIASE_1 (PS00170, 55aa-72aa). The results of gene ontology (GO) analysis indicated that protein folding (GO:0006457), protein peptidyl-prolyl isomerization (GO:0000413), and peptidyl-prolyl cis–trans isomerase activity (GO:0003755) were enriched. Further analysis revealed that the *PgCyP* protein contains a conserved sequence (vsgkplh: 48aa-54aa) (Figure 2), the active site of PPIase (R62, F67, and H133), and the binding site for cyclosporin A (w128). These results show that the *PgCyP* protein has PPIase activity and functions by binding with CsA. Furthermore, it can catalyze the cis–trans isomerization of the peptide proline and plays a key role in post-translational protein processing.

**Figure 3:**
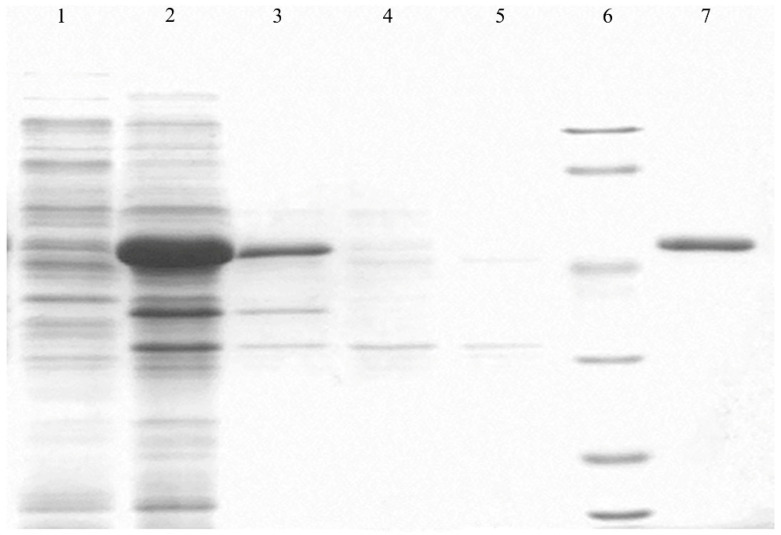
Expression and purification of pGEX-6p-1-PgCyP. Lane 1, soluble protein fraction; Lane 2, insoluble protein fraction; Lane 3, crude protein solution; Lane 4, purification flow-through fraction; Lane 5, after protein purification; Lane 6, marker; Lane 7, purification elution fraction with 100 mM imidazole.

**Figure 4:**
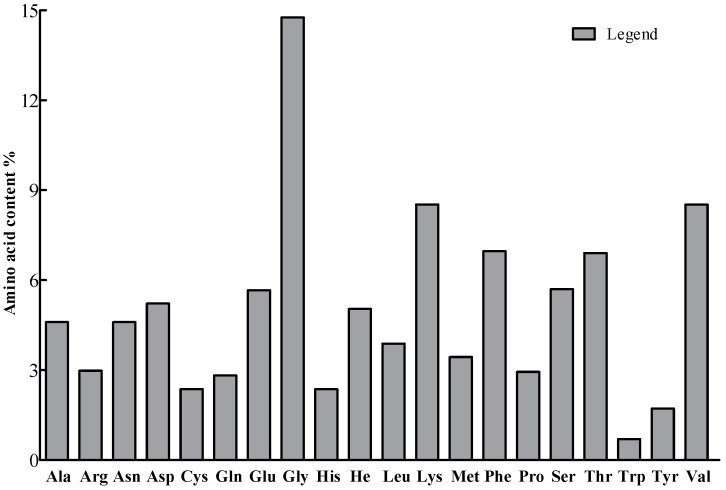
Analysis of amino acid species. Ala: alanine, Arg: arginine, Asn: asparagine, Asp: aspartic, Cys: cysteine, Gln: glutamine, Glu: glutamic, Gly: glycine, His: histidine, He: isoleucine, Leu: leucine, Lys: lysine, Met: methionine, Phe: phenylalanine, Pro: proline, Ser: serine, Thr: threonine, Trp: tryptophan, Tyr: Tyrosine, and Val: valine.

The protein purification electropherogram is shown in Figure 3. The relative molecular weight of PgCyP is 187.11 kDa, and its theoretical isoelectric point is 7.67. There are 19 negatively charged amino acid residues (Asp and Thr) and 20 positively charged amino acid residues (Arg and Lys) (Figure 4). On the basis of this, the PgCyP protein is classified as a basic protein. Among the amino acid residues in the PgCyP protein, Gly accounts for the highest proportion, followed by Lys, Val, Phe, and Thr; Leu accounts for the lowest proportion. In all, nine amino acid residues account for over 5%.

#### 2.2.2. Analysis of the Hydrophobicity/Hydrophilicity of the PgCyP Protein

**Figure 5:**
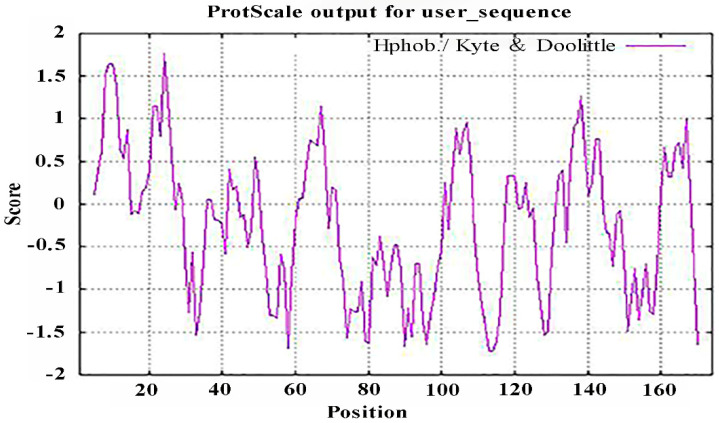
Analysis of the hydrophilicity/hydrophobicity of the PgCyP protein.

It is important to study the biological functions of proteins to analyze their hydrophobicity/hydrophilicity. The hydrophobicity and hydrophilicity of the PgCyP protein were analyzed using ProtScale, and the results (Figure 5) revealed that there were two highly hydrophobic amino acids in the anterior region of the N-terminal. Therefore, we believe that the PgCyP protein is a hydrophobic protein.

#### 2.2.3. Analysis of the Advanced Structure of the PgCyP Protein

**Figure 6:**
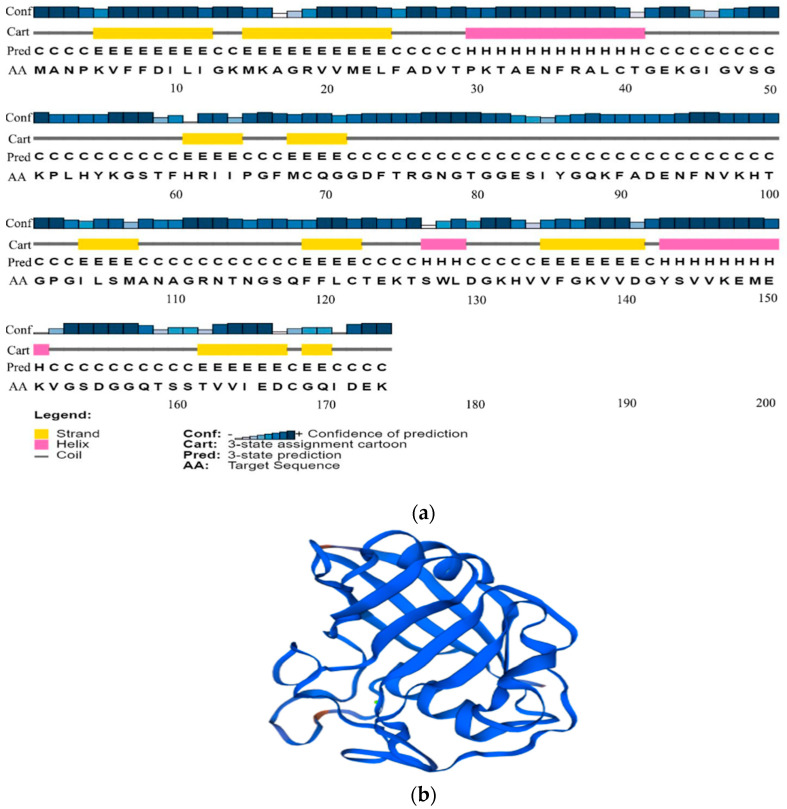
Structure prediction results of the PgCyP protein. (**a**) Protein secondary structure prediction. Different colors represent different types of secondary structures (yellow: beta-strands; red: alpha helix; and gray: random coil). (**b**) Tertiary structure prediction of the PgCyP protein.

The secondary structure of the PgCyP protein consists of three α-helixes (about 11.54%), 9 β-sheets (about 34.62%), and 14 random curls (about 53.84%). The tertiary structure is a barrel conformation. Therefore, it can catalyze the rate-limiting step in the protein folding process, that is, the cis–trans isomerization of peptidylprolyl (Figure 6).

#### 2.2.4. Analysis of the Cis-Acting Element of the Promoter of the *PgCyP* Gene

To investigate the PgCyP protein’s function, an analysis of the promoter region was performed (Table 1). Specifically, the 163 bp region upstream of the gene’s ATG codon, namely the gene promoter region, was analyzed. The results of the analysis indicated that the PgCyP protein contained LTR, ABRE, and G-BOX, which means that the PgCyP protein may play a role in plant hormone signal transduction, the response of plants to environmental stress, and light signal transduction.

#### 2.2.5. Prediction of Signal Peptides and the Transmembrane Structure

**Figure 7:**
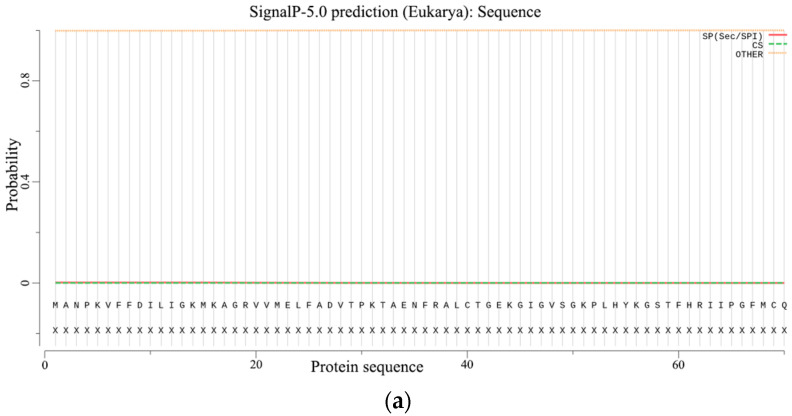

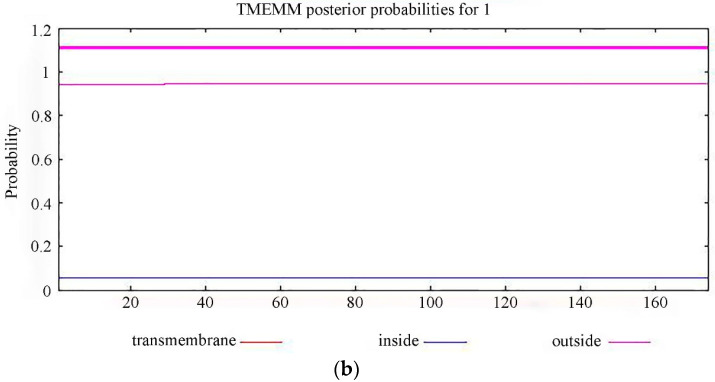
Prediction of the signal peptide (**a**) and transmembrane structure (**b**) of the PgCyP protein.

The prediction of signal peptides and the transmembrane structure is an important basis for distinguishing whether a protein is a secretory protein. Without a signal peptide, the PgCyP protein is not a secretory protein (Figure 7a). The total probability of N- in the PgCyP protein was 0.05539, and all the amino acid residues were outside the biofilm (Figure 7b). This indicates the absence of transmembrane structure in the PgCyP protein, suggesting that this protein is located within the cytoplasm.

#### 2.2.6. Analysis of the Phylogenetic Tree of the PgCyP Protein

**Figure 8:**
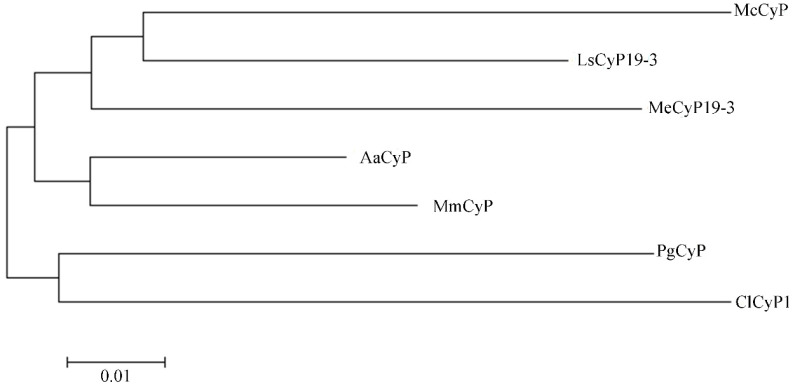
Phylogenetic tree of PgCyP.

A phylogenetic tree was constructed with similar sequences (PWA85506.1 (Aa*CyP*), KAD6796696.1 (Mm*CyP*), XP_021624038.1 (Me*CyP*19-3), AAV48823.1 (Cl*CyP*1), OVA02441.1 (Mc*CyP*), and XP_023755431.1 (Ls*CyP*19-3)) and the sequence of the PgCyP protein (Figure 8). In the phylogenetic tree, the PgCyP protein occupied a distinct position in comparison with others and exhibited a closer relationship with Cl*CyP*1 (AAV48823.1). These results indicate that PgCyP is a very conservative protein in evolution.

#### 2.2.7. Prediction Results of the Phosphorylation Sites of the PgCyP Protein

**Figure 9:**
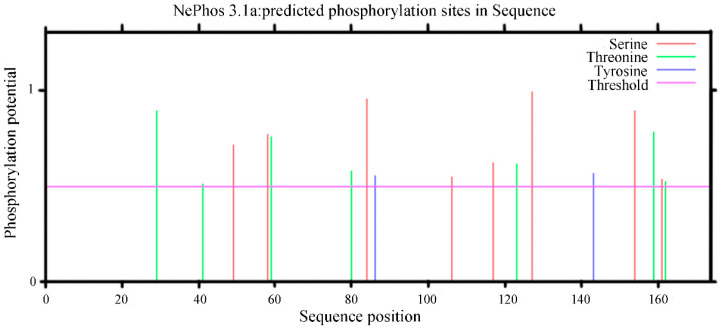
Prediction of the phosphorylation sites of the PgCyP protein in *P. ginseng*.

Protein phosphorylation plays an important role in the process of cell signal transduction; therefore, the prediction of the phosphorylation site of the PgCyP protein is helpful in predicting the function of protein phosphorylation. The NetPhos3.1 analysis results (Figure 9) revealed nine phosphorylation sites in the PgCyP protein, comprising four serine phosphorylation sites, three threonine phosphorylation sites, and two tyrosine phosphorylation sites.

### 2.3. Anti-P. cactorum Activity of the Recombinant PgCyP Protein

#### 2.3.1. Effect of the Recombinant PgCyP Protein on the Spores

**Figure 10:**
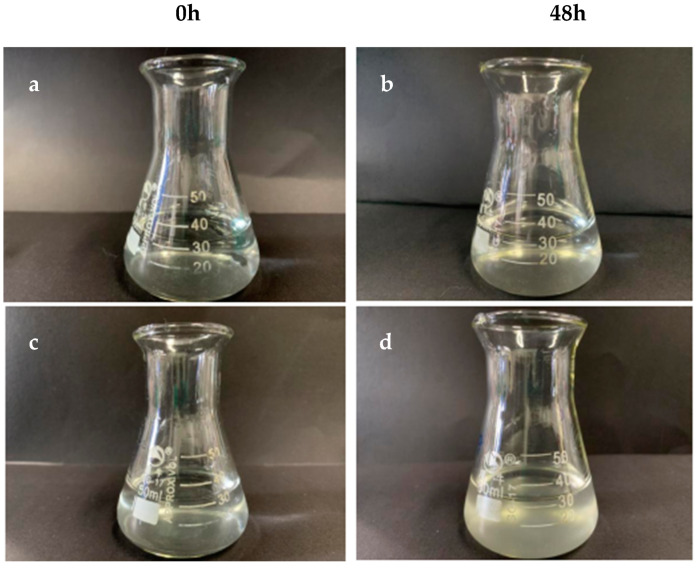
Resistance of protein extracts to conidia of *P. cactorum*. (**a**,**b**) Spore suspension in PBS medium. (**c**,**d**) Purified protein and spore suspension in PBS medium.

**Figure 11:**
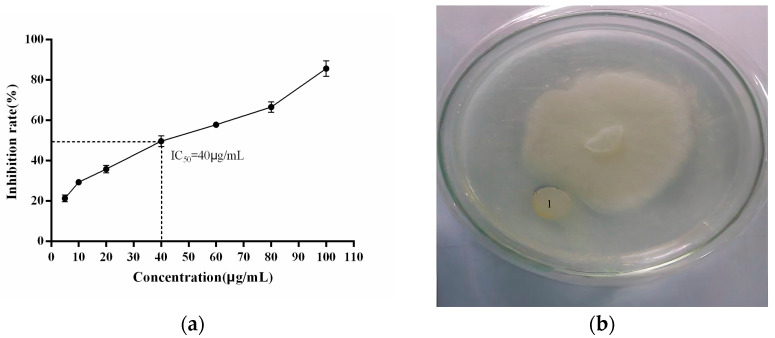
Effects of different concentrations of PgCyP on *P. cactorum* and its inhibitory effect: (**a**) effects of different concentrations of PgCyP on *P. cactorum* and (**b**) inhibition zone of *P. cactorum(1 stands for inhibition zone of P. cactorum)*.

**Figure 12:**
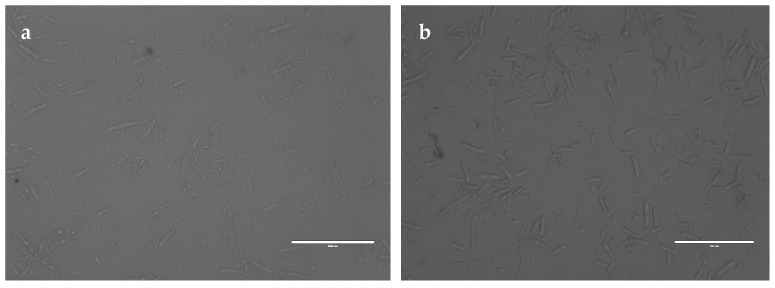
Effect of PgCyP on conidia germination of *P. cactorum*: (**a**) experimental group and (**b**) control group. Scale bar: 100 μm.

To analyze the interaction between the recombinant PgCyP protein and *P. cactorum*, the recombinant PgCyP protein was co-cultured with the spores of *P. cactorum* for 48 h. As shown in Figure 10, the culture medium of the control group (without a recombinant protein) became turbid, while that of the experimental group remained clear. Additionally, Figure 11a shows that the semi-inhibitory concentration of the recombinant PgCyP protein for *P. cactorum* was 40 μg/mL, which was chosen for subsequent experiments. The results of co-culture experiments between pathogenic fungi and recombinant proteins showed that the recombinant proteins had a significant inhibitory effect on *P. cactorum*. With the massive growth of hyphae, a clear inhibition zone appeared around the surface of the culture medium where the recombinant protein drug-sensitive tablets were dropped. By contrast, no inhibition zone was observed around the control group (the empty carrier protein drug-sensitive tablets), as shown in Figure 11b. Additionally, Figure 12 shows that the spores of *P. cactorum* in the control group germinated normally, and the mycelium also grew normally. However, only a few spores of *P. cactorum* in the experimental group germinated, and the spores were contracted; the mycelia became shorter and had more branches. All the above revealed that the recombinant PgCyP protein effectively inhibits spore germination and normal mycelial growth in vitro.

#### 2.3.2. Effect of the Recombinant PgCyP Protein on the *P. cactorum* Resistance

**Figure 13:**
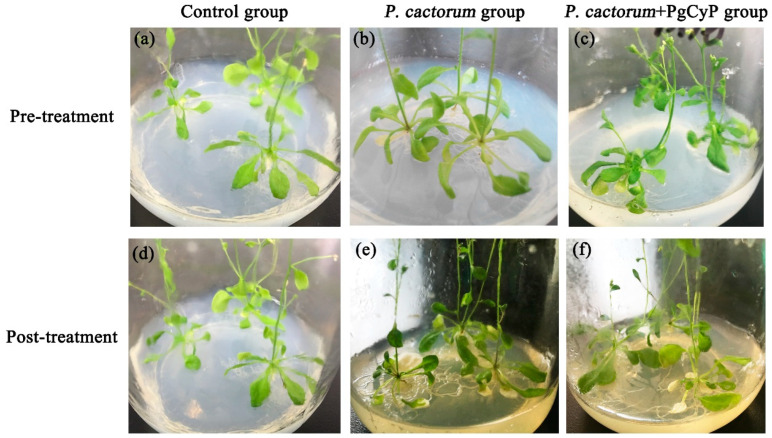
Inhibition of fungal infection by the PgCyP protein. (**a**,**d**): Control group. (**b**,**e**): *P. cactorum* group. (**c**,**f**): *P. cactorum* + PgCyP group.

In this research, the *PgCyP* gene was transferred into *A. thaliana*, and its effect against the infection of *P. cactorum* was studied (Figure 13). The *A. thaliana* control group grew well, the color of the leaves was normal, and the disease index was 0%. However, the *A. thaliana* seedlings in group B, which was infected with spores of *P. cactorum*, were almost dead, the color of the root turned yellowish to yellowish brown, most of the leaves withered and turned white, and the disease indices were 94%, 90%, and 97%, respectively. The *A. thaliana* seedlings in group C, which was treated with the spores of *P. cactorum* and the recombinant PgCyP protein, grew normally, with only a few leaves turning white, and the disease indices were 17% and 0.39%, respectively. These results suggest that the PgCyP recombinant protein has a positive effect on the resistance of *Arabidopsis* to the infection of *Phytophthora* spores. Furthermore, the PgCyP protein can improve the resistance of *Arabidopsis* to *P. cactorum* by inhibiting spore germination and hyphal growth, and it is expected to become a new green biological pesticide.

## 3. Discussion

Cyclophilin is widely present in various organisms. It plays an important role in regulating cell signaling pathways [27,28], mediates the immunosuppressive effect of cyclosporine A, and reduces various parasitic infections [29]. Cyclophilin also binds to DNA and is involved in regulating cell apoptosis [18], promoting protein folding, functioning as molecular chaperones [30,31], and performing various stress-regulating functions [32].

The antifungal activity of cyclophilins may be related to their role in fungal stress response. In this paper, the full-length gene of ginseng cyclophilin was obtained from an established ginseng cDNA library. Using pGEX-6P-1 as the expression vector, a prokaryotic expression system for ginseng cyclophilin was successfully constructed and implemented in *E. coli* for protein fusion expression. The *PgCyP* gene contains a 522 bp open reading frame, which can encode 174 amino acids. The predicted theoretical molecular weight of the protein is about 19 KDa, which is consistent with the known molecular weight of cyclophilin. Ginseng diseases mostly occur during the rainy season (July–September). By analyzing the transcriptional expression levels of the gene in ginseng roots, stems, and leaves, it was found that the ginseng cyclophilin gene is highly expressed in the red fruit stage of ginseng roots. Therefore, we hypothesize that the expression level of the ginseng cyclophilin gene is closely related to the period when ginseng is susceptible to disease. The experimental results of the antifungal activity of ginseng cyclophilin protein showed that a low concentration (μM) of PgCyP protein had a strong inhibitory effect on the growth of *Phytophthora* ginseng. In the PPIase activity test, PgCyP had noticeable peptide coamyl cis–trans isomerase activity compared with the blank control. On the basis of the above experiments, it was proven that ginseng cyclophilin protein has antifungal activity. To date, full-length genes of cyclophilins have been obtained from a variety of plants, and more in-depth research has been conducted on their functions. For instance, researchers have proven that the cabbage cyclophilin C-CyP is effective against *Botrytis cinerea*, *Trichoderma harzianum*, green mold, and *Trichoderma* spp. Additionally, fungi such as *Rhizoctonia solani*, *Fusarium solani*, and *Fusarium oxysporum* have been shown to have inhibitory effects [20]. However, this experimental study found that ginseng cyclophilin protein has an inhibitory effect on the growth of *Phytophthora*.

At present, many antifungal proteins are used in genetic engineering and breeding. Cyclophilin, a kind of antifungal protein, has also been used in plant transgenic research. However, there are no specific reports on its specific mechanism in the antifungal activity of ginseng. This study shows that ginseng cyclophilin protein can effectively inhibit the growth of *Phytophthora* in vitro. To this end, transgenic technology can be used to express the ginseng cyclophilin gene in plants, which can help us better determine the role of cyclophilin in the antifungal process and provide a theoretical basis for biological disease control.

## 4. Materials and Methods

### 4.1. Materials

Five-year-old P. ginseng plants were collected from a ginseng planting region in Fusong County, Jilin Province, and were identified by the Department of Traditional Chinese Medicine Identification of Changchun University of Traditional Chinese Medicine as conforming to the provisions of the Chinese Pharmacopoeia (2020 Edition, Part I). The cloning vectors DH5α and pMD-18T were bought from BaoRi Biotechnology Co., Ltd. (Zhuhai, China), and *P. cactorum* was identified and provided by Prof. Yanglimin from the College of Traditional Chinese Medicine, Jilin Agricultural University. Col-0 Arabidopsis thaliana was kept at the Bioengineering Laboratory of the Changchun University of Chinese Medicine. The ginseng and Arabidopsis thaliana involved in this article were obtained legally and properly licensed.

### 4.2. Reagents and Instruments

The media and instruments used in the study included potato dextrose agar media (1000 mL each, which contained 1000 mL of potato extract, 20 g of glucose, and 18 g of agar powder); a QHX-400BS-III artificial climate box (Shanghai Xinmiao Medical Device Manufacturing Co., Ltd., Shanghai, China); a nucleic acid electrophoresis instrument (Mupid, Kyoto); and a CFX96 TouchTM fluorescence quantitative PCR detection system (Bio-Rad Laboratories, Inc., Hercules, CA, USA).

### 4.3. Obtaining and Cloning of the PgCyP Gene

Total mRNA was extracted using an improved TRIzol method. *Panax ginseng* cDNA was synthesized through reverse-transcription PCR. To clone the PgCyP gene, two specific primers were designed according to the reported DNA sequence [24]. The primer design is shown in Table 2. The RT-PCR reaction conditions were as follows: 94 °C for 3 min, followed by 35 cycles at 94 °C and 55 °C for 30 s and 72 °C for 1 min, and then a final extension at 72 °C for 5 min. The PCR products were purified and ligated into the vector pMD-18T. The recombinant plasmids were then transformed into DH5α competent cells, which were plated on coated plates, and monoclonal colonies were subsequently selected. The plasmid was extracted and sent to Sangong Bioengineering (Shanghai) Co., Ltd. (Shanghai, China). The sequencing results were compared with DNAMAN for subsequent bioinformatics analysis.

### 4.4. Bioinformatics Analysis

The physical and chemical properties, as well as types of amino acids, were analyzed using ExPASy-ProtParam, and the isoelectric point was evaluated with the same tool (https://web.expasy.org/protparam/) (accessed on 25 May 2024) [25]. InterPro, an integrated database of protein structural domains and functional sites, (https://www.ebi.ac.uk/interpro/result/InterProScan/iprscan5-R20231118-050315-0332-41996300p1m/) (accessed on 25 May 2024), was used for further analysis. The hydrophobicity and hydrophilicity of the PgCyP protein were analyzed with ExPASy-ProtScale. The second structure of the PgCyP protein was analyzed with UCL-CS Bioinformatics: PSIPRED, and the spatial structures of the PgCyP protein were analyzed with Phyre2 [26]. The protein’s signal peptide was analyzed using the SignaIP-5.0 Server [33]. Furthermore, the protein’s transmembrane structure was analyzed with TMHMMServerv 2.0 (http://www.cbs.dtu.dk/services/TMHMM-2.0/) (accessed on 25 May 2024), and the protein phosphorylation sites were analyzed with NetPhos (http://www.cbs.dtu.dk/services/NetPhos/) (accessed on 25 May 2024). The amino acid sequences were blasted against NCBI (https://blast.ncbi.nlm.nih.gov/Blast.cgi) (accessed on 25 May 2024), and a phylogenetic tree was constructed using highly similar sequences (Artemisia annua: PWA85506.1, Mikania micrantha: KAD6796696.1, Manihot esculenta: XP_021624038.1, Codonopsis lanceolate: AAV48823.1, Macleaya cordata: OVA02441.1, Lactuca sativa: XP_023755431.1, and *Panax ginseng*: ANA12002.1) with MEGA 7.0. The gene’s intron and exon were analyzed with Exon-Intron Graphic Maker. The cis-acting element of the promoter sequence of the *PgCyP* gene was analyzed with Plant Care.

### 4.5. Expression and Purification of PgCyP

PgCyP was purified through a Ni affinity chromatography column and then freeze-dried to a concentrate. The protein was further diluted to 10 mg·mL^−N^ and filtered with a 0.22 μm filter [24,34].

### 4.6. Preparation of the Spore Suspension

The mycelia of *P. cactorum* were aseptically transferred to a conical bottle containing sterile glass beads and a sterile glass plate. Fungal conidia were cultured in a PDB medium at 25 °C under constant shaking at 150× *g* for 3–4 d. Subsequently, the spores were filtered with 8 layers of sterile gauze. The final conidial suspension of 2.5 × 10^7^ cells/mL was prepared in sterile water with a hemocytometer [35].

### 4.7. Experimentation on the Anti-Phytophthora cactorum Activity of the Recombinant Protein

#### 4.7.1. Effects of Different Concentrations of PgCyP on *P. cactorum*

Fifty-milliliter triangular conical flasks were autoclaved at 121 °C, after which 20 mL of the PDB culture medium was added to each flask. Then, small pieces of protozoa from mycobacterium Petri dishes were inoculated into the PDB culture medium, and the flasks were incubated for 3 d at 28 °C and 150 r/min. After incubation, the spore solution was filtered through eight-layer gauze, collected, and diluted with sterilized water to achieve the desired concentration (10^4^ spores/mL). Subsequently, 80 μL of the diluted spore solution was transferred to a 96-well plate. Recombinant protein concentrations of 5, 10, 20, 40, 60, 80, and 100 μg/mL were tested, and three parallel experiments were conducted. The absorbance value of each well was recorded at 595 nm using an enzyme labeling instrument, and the growth curve was plotted.

#### 4.7.2. *P. cactorum* Activity Inhibition

A sterile filter paper piece with a temperature of 121 °C and a diameter of 6 mm was placed at an appropriate distance from the edge of the mycelium, after which 40 μg/mL of recombinant protein was added dropwise to fully infiltrate the drug-sensitive paper piece. For the control, 15 μL (the carrier protein pGEX-6p-1 (concentration: 1.32 μg/μL)) was used. The Petri dish was then sealed with a sealing film, placed upside down in a mold incubator, and cultured at 28 °C for 3 d. According to the diameter of the inhibition zone, the sensitivity of the *P. cactorum* to the PgCyP protein was determined.

#### 4.7.3. Effect of the Recombinant Protein on Spores

An optical microscopic observation conducted according to Karri’s method [36] confirmed that the recombinant PgCyP protein inhibited the growth of *P. cactorum*. The spore suspension of *P. cactorum* (4 mL, containing 1.8 × 10^5^ CFU/mL) and recombinant purified protein (5 μL 40 mg/mL) were co-cultured in PDB (40 mL) at 25 °C for 48 h. After that, 200 μL of the culture medium was used for observing the germination and growth of the spores.

#### 4.7.4. Effect of the Recombinant Protein on the *P. cactorum* Resistance of *A. thaliana*

A. thaliana seeds were surface-sterilized with 75% ethanol for 5 min and then with 2% sodium hypochlorite for 7 min. Finally, all the seeds were placed onto Murashige and Skoog (MS) plates and transferred to the phytotron after being cultured at 4 °C for 48 h for vernalization treatment. According to the plant infection method described by Xiao and Chye [37], 24-day-old wild-type *A. thaliana* seedlings showing good growth were randomly divided into three groups: group A (non-treatment), group B (sprayed with spore suspension (1.8 × 10^7^ CFU/mL) and sterilized water), and group C (sprayed with spore suspension (1.8 × 10^7^ CFU/mL) and recombinant purified protein (40 mg·mL^−1^)). After 7 days of the treatment, the disease index and growth of the three groups were observed and recorded.

The disease index is a comprehensive index to calculate the infection rate and the number of infected leaves [38]. It is divided into 5 levels depending on the rotten area of the leaf: level 0 indicates a lack of symptoms; in level 1, chlorotic reaction and hypersensitive response (HR) are present around the infected area; in level 2, less than 50% of the leaf area is rotten; in level 3, approximately 50–75% of the leaf area is rotten; and in level 4, the leaf and petiole are rotten and chlorotic.

$$\mathrm{Disease}\;\mathrm{index}=\sum \left({SL}_{i}\times L_{i}\right)/\left({SL}_{ts}\times L_{m}\right)\times 100\%$$

where Li is the level, SLi is the sum of the leaves in level i, SLts is the total sum of the leaves in all the levels, and Lm is the maximum level.

### 4.8. Statistical Analysis

All assays were performed in triplicate, and the results are expressed as means ± standard deviation. Statistical significance was analyzed using one-way ANOVA followed by the Tukey–Kramer multiple comparisons test using GraphPad Prism 9.3.0 and SPSS 19.0 (IBM, Armonk, NY, USA). Differences with values of *p* ≤ 0.05, *p* ≤ 0.01, and *p* ≤ 0.001 were considered statistically significant to varying degrees.

## 5. Conclusions

The results of this study not only provide a theoretical basis for the biological control of ginseng rust rot but also provide a basis for the application and development of a ginseng CYP protein in biological pesticides. The length of the *PgCyP* gene is 525 bp in the CDS region, and it encodes 174 amino acids, including the conserved cyclophilin sequence vsgkplh (48aa-54aa), the active site of PPIase (R62, F67, and H133), and the binding site for cyclosporine A (w128), with the peptidyl proline cis–trans isomerase activity and CSA binding. The PgCyP protein has no signal peptide or transmembrane domain, but it has good hydrophobicity and contains nine phosphorylation sites. The promoter region of PgCyP mainly contains the low-temperature environmental stress element (LTR), the hormone (ABA) regulatory element (ABRE), and the light response element (G-box). In the phylogenetic tree, *PgCyP* (ci Cy P1) is very close to the ci Cy P1 of *Codonopsis*, indicating that the PgCyP protein belongs to a conserved protein family. The inhibitory effect of the PgCyP protein on *P. cactorum* may be related to the peptidyl proline cis–trans isomerase activity of PgCyP. Furthermore, the PgCyP protein can also inhibit the growth of *P. cactorum* and is expected to become a new green biopesticide.

## Figures and Tables

**Table 1:** Cis-acting elements predicted in the promoter sequence of the PgCyP gene.

Element	Type	Function	Sequence	No.
ABRE	Hormone regulatory element	ABA-responsive cis-acting element	ACGTG	1
CAAT-box	Upstream elements of the genome	Gene regulatory region	CAAAT	2
CAAT-box	Upstream elements of the genome	Gene regulatory region	CAAT	1
G-Box	Light regulatory element	Light response relative cis-acting element	CACGTT	1
G-box	Light regulatory element	Light response relative cis-acting element	TAACACGTAG	1
LTR	Stress inducing element	Low-temperature response relative cis-acting element	CCGAAA	1
TATA-box	Upstream elements of the genome	Core promoter element	ATATAT	1
TATA-box	Upstream elements of the genome	Core promoter element	ATATAA	1
TATA-box	Upstream elements of the genome	Core promoter element	TATA	2

**Table 2:** Primers for the gene expression analysis of PgCyP.

Gene	Accession Numbers	Primers’ Sequence (5’–3’)
*CyP*	ANA12002.1	F: CGGATCCATGGCCAACCCAA
		R: ATTTGCGGCCGCGTGGTGGTGGTGGTGGTGCTCGAG
*V-ATP*	KF699328	F: AAGAGTGCCATTGGTGAGG
		R: CCTTGAGCGACAAACTTCC
*CyP*	KF699321	F: CAGGCAAAGAAAAAGTCAAGTG
		R: AAAGAGACCCATTACAATACGC

## Data Availability

The original contributions presented in the study are included in the article. Further inquiries can be directed to the corresponding author.
